# Clinical and economical impacts of guideline implementation by the pharmaceutical care unit for high cost medications in a referral teaching hospital

**DOI:** 10.1186/s12913-018-3627-3

**Published:** 2018-10-24

**Authors:** Afsaneh Vazin, Iman Karimzadeh, Razieh Karamikhah, Zahra Oveisi, Samaneh Mohseni, Maryam Keykhaee, Fatemeh Roshanfard, Elaheh Sabet, Asal Zargari-Samadnejad

**Affiliations:** Department of Clinical Pharmacy, Faculty of Pharmacy, Shiraz University of Medical Sciences, Shiraz, Iran

**Keywords:** Albumin, Intravenous pantoprazole, Intravenous immune globulin, Direct cost, Pharmaceutical care unit, Guideline implementation

## Abstract

**Background:**

Irrational drug use is a global health challenge in all healthcare settings, such as hospitals. This study evaluated the impact of an intervention by the pharmaceutical care unit on the use pattern of high-value medications and their direct costs in a referral hospital.

**Methods:**

This interventional, prospective study was carried out in clinical wards of Namazi Hospital (Shiraz University of Medical Sciences) during six months from May 2015 to October 2015. Clinical pharmacists completed the checklists for albumin, intravenous (IV) pantoprazole, and IV immune globulin (IVIG), as three high-cost medications. When ordering these medications, the physicians were asked to complete the checklists. Then, trained pharmacists examined the checklists, based on the clinical and paraclinical conditions.

**Results:**

The total number of administered medications and their relative cost decreased by 50.76% through guideline implementation; the difference was significant (*P* <  0.001). In addition, the direct cost of albumin and IV pantoprazole significantly decreased (55.8% and 83.92%, respectively). In contrast, the direct cost of IVIG increased by 40.9%. After guideline implementation, the monthly direct cost of all three medications decreased by $77,720 (55.88%). The all-cause in-hospital mortality rate did not change significantly due to the intervention. The median length of hospital stay was six and seven days, respectively in the pre- and post-intervention periods.

**Conclusion:**

Based on the findings, implementation of guidelines by the pharmaceutical care unit caused a significant reduction in albumin and IV pantoprazole consumption and reduced their direct costs in a referral center in Iran.

**Electronic supplementary material:**

The online version of this article (10.1186/s12913-018-3627-3) contains supplementary material, which is available to authorized users.

## Key points

• Guideline implementation by pharmacists significantly reduced the direct cost of albumin and IV pantoprazole.

• Guideline implementation was not an effective method for decreasing the direct cost of IVIG.

• Direct supervision of pharmaceutical care units can improve the pattern of medication use and cost-saving strategies.

## Background

Irrational drug use is a global challenge in all healthcare settings, such as hospitals. Furthermore, prescribers in the community continue inappropriate hospital prescriptions. This is a concerning issue since medical as well as financial resources are limited, particularly in developing countries [1]. In developing countries, although drugs constitute up to 40% of the healthcare budget, a large number of people may not have adequate access to the most basic or essential medicines [2].

The World Health Organization has suggested different administrative, educational, and regulatory strategies to improve the drug use pattern, including continuous healthcare team training, standard clinical practice guidelines, and drug utilization evaluation [1]. Implementation of clinical practice guidelines based on a pharmaceutical cost-containment program enhances patient safety through minimization of adverse effects and drug interactions, reduction of inappropriate medication prescriptions, controlling resistance of bacterial pathogens to antimicrobial agents, decreasing costs, management of medication supplies, improving the quality of healthcare, and promotion of patient satisfaction [3, 4].

There is relatively limited data on the pharmacists’ role in both promoting adherence to guidelines and improving clinical and/or economic outcomes [5–7]. This study aimed to assess the impact of interventions by pharmaceutical care units on the direct cost and use pattern of three high-cost medications in a referral hospital in Southwest of Iran.

## Methods

### Study setting

This prospective, interventional study (Iranian Registry of Clinical Trial ID: IRCT20161010030246N2) was performed during 6 months from May 2015 to October 2015 in all clinical wards of Namazi Hospital. This period was considered as the post-intervention phase, while the period from April 2014 to September 2014 was identified as the pre-intervention phase. Namazi Hospital, affiliated to Shiraz University of Medical Sciences (Shiraz, Iran) is a general tertiary referral center with 50 wards and nearly 1000 beds. No limitations were considered in selecting 50 clinical wards during the post-intervention phase. The study was approved by the hospital Medical Ethics Committee and Institutional Review Board, and written informed consents were obtained from patients or their family members.

### High-cost medications

The database of the automated Hospital Information System regarding prescribed medications in 2014 was analyzed via ABC analysis described elsewhere [1]. Considering the total number of medicines consumed and their relative cost, albumin, intravenous (IV) pantoprazole, and IV immunoglobulin (IVIG) were the top three medications, accounting for the largest proportion of the hospital budget. Overall, 21.3%, 19.5%, and 13.7% of the pharmaceutical budget of the hospital in 2014 were spent for albumin, IVIG, and IV pantoprazole, respectively. The costs were converted to United States dollars at the real-time conversion rate.

### Indication checklist

The clinical pharmacists designed an indication checklist draft for the selected drugs by exploiting relevant references and textbooks, such as online UptoDate, as well as Micromedex, American Society of Health System Pharmacists guidelines [8], and Applied Therapeutics: The Clinical Use of Drugs by Koda-Kimble and Young [9]. Subsequently, checklists were reviewed by the hospital medical team, consisting of medical experts with different relevant specialties and sub-specialties (including internists, rheumatologists, nephrologists, gastroenterologist, hematologists, cardiologists, surgeons, and neurologists), and their comments were implemented. The final version of indication checklist consisted of the following items: 1) demographics and related clinical and paraclinical data of the patient, 2) brief description of each indication, 3) medication order, 4) physician and pharmacist comments, and 5) final decision (approved or disapproved). Indication checklists for albumin, IVIG, and IV pantoprazole are provided in Additional file 1.

### Guideline implementation

During the pre-intervention phase, wards were allowed to receive their requested medications from the hospital pharmacy according to physician team prescriptions without any limitations. Prescriptions in this period were generally based on both clinical and paraclinical conditions of patients and also physicians’ preferences with no data regarding the rate of inappropriate use.

After coordination with the head of different hospital departments, the physicians’ teams (including attending, fellowships, or residents) were requested to complete the indication checklist form when ordering albumin, IV pantoprazole, and IVIG. The checklists were examined by one of the five general pharmacists based on the clinical conditions and paraclinical data of patients during morning and afternoon shifts on working days (Saturday to Thursday).

General pharmacists were authorized to either approve or disapprove the indication checklist forms under the supervision of clinical pharmacists. Controversial or complicated cases (*n* = 124) were discussed in regular joint meetings of senior physicians and clinical pharmacists. In other words, the early acceptance rate of guideline implementation in our hospital by the physicians’ team was 83.04%. Possible inter- and intra-individual variations between general pharmacists were minimized by the direct and regular supervision of a senior clinical pharmacist. No specific educational course regarding the appropriate utilization of studied medications was designed or implemented for the physicians’ team in the pre- and post-intervention periods.

**Fig. 1:**
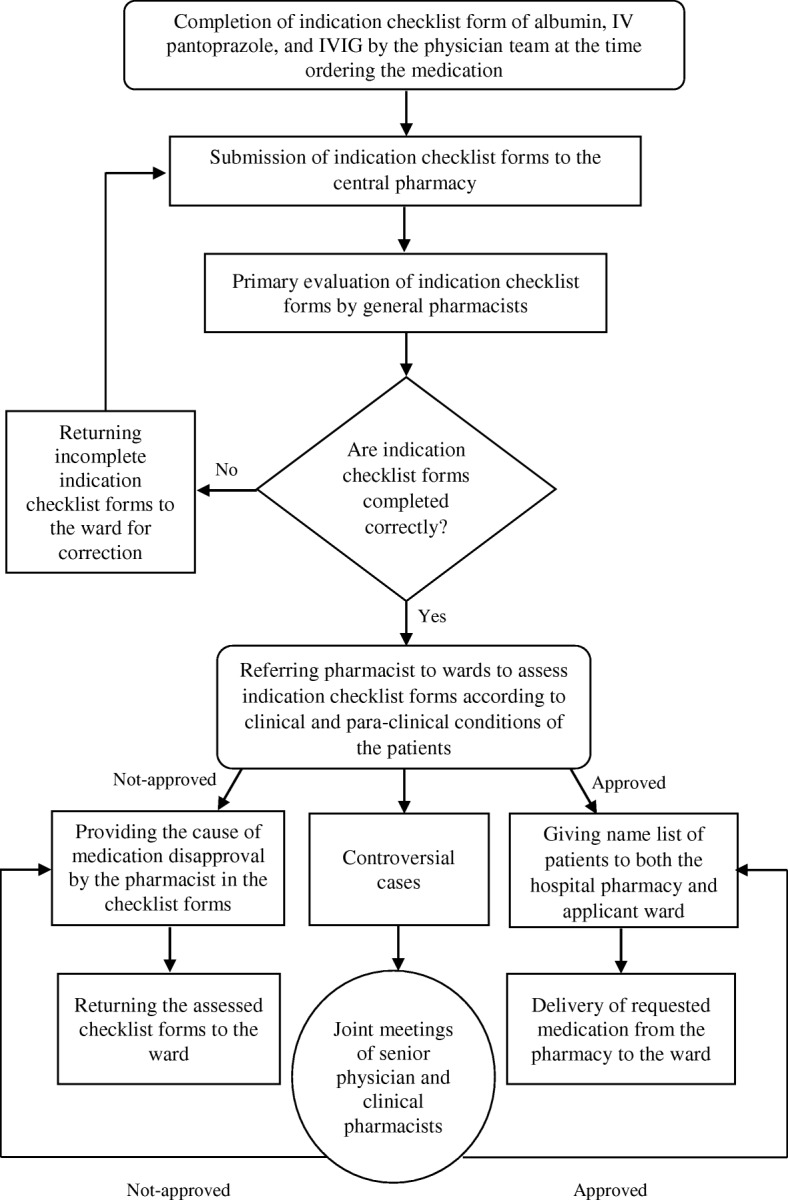
Flow chart of pharmaceutical care unit guideline for the use of albumin, intravenous pantoprazole, and IVIG

Daily lists of names of patients, whose medications were approved, were prepared by pharmacists and given to both the hospital pharmacy and wards. The wards were permitted to take their medications in emergency conditions (e.g., IV pantoprazole for active upper GI bleeding) without the approval of the pharmaceutical care unit during public holidays. In such conditions, the indication checklist forms were checked by the pharmacists at the earliest possible time after the night or holiday shifts. The flow chart of pharmaceutical care unit guidelines is depicted in Fig. 1.

### Data collection

Demographic data (including age and sex), admission ward, prescription number and direct cost of each studied medication, and general indices of clinical outcomes (hospital length of stay [LOS] and all-cause in-hospital mortality) were extracted for all patients in both pre- and post-intervention periods from the Hospital Information System. It is worth noting that individuals who received any of the studied medications in the pre-intervention period were considered eligible for inclusion. The cause of disapproved requests for each medication in the post-intervention period was also recorded. In order to eliminate the probable effect of price changes between pre- and post-intervention periods, costs of studied medications in April 2014 (price stability) were considered for all relevant calculations.

### Statistical analysis

Categorical variables are expressed as percentage. The normal distribution of continuous variables was examined using Kolmogorov-Smirnov test. Continuous data with and without a normal distribution are expressed as mean ± SD and median (interquartile range), respectively. To determine the relationship between categorical variables, Chi square or Fisher’s exact test was performed (if more than 25% of the categories have frequencies below five). Also, for evaluating parametric and non-parametric continuous variables, independent t test and Mann-Whitney test were applied, respectively.

Univariate logistic regression analysis was used to separately assess the plausible associations between each independent variable (age, sex, admission ward, and intervention) and in-hospital mortality as the dependent variable. Independent variables with *P* <  0.05 were entered in the multivariate logistic regression model. *P* < 0.05 was considered significant for the analytical tests. All analyses were performed in SPSS version 20 (IBM Company, New York, NY, USA).

## Results

### Patient characteristics

In the pre- and post-intervention phases, 4946 and 4895 patients were included, respectively. The patients’ characteristics in the pre- and post-intervention periods are listed in Table 1. More than half (58%) and three-fifth (60.29%) of individuals were males in the pre- and post-intervention phases, respectively. Sex distribution was comparable between the two groups. In contrast, the median interquartile range (IQR) of age was significantly higher (*P* = 0.002) in the pre-intervention period [50 (38)] than the post-intervention phase [48 (46)]. Except for pediatrics (*P* < 0.0001) and plastic surgery wards (*P* = 0.001), distribution of patients in all wards was comparable between the pre- and post-intervention periods. The hospital bed occupancy rate in the pre- and post-intervention phases (92.8% and 92%, respectively) did not differ significantly (*P* = 0.639).

**Table 1:** Patients characteristics in the pre- and post-intervention periods

Variable	Pre-intervention group (*n* = 4946)	Post-intervention group (*n* = 4895)	*p*
Sex, *n* (%)			
Male	2901 (58)	2951 (60.29)	0.097
Female	2045 (42)	1944 (39.71)	
Age			
Median, year (IQR)	50 (38)	48 (46)	0.002
Range	1.2 month-99 years	1.2 month-98 years	
Hospital wards, *n* (%)			
Internal	886 (17.9)	817 (16.8)	0.095
General surgery	279 (5.6)	264 (5.4)	0.52
Neurosurgery	284 (5.7)	241 (4.9)	0.061
Cardiac surgery	139 (2.8)	112 (2.3)	0.088
Urology	60 (1.2)	68 (1.4)	0.480
Intensive care	814 (16.4)	860 (17.73)	0.261
Pediatrics	1026 (20.7)	1195 (24.64)	< 0.0001
Emergency	1180 (23.8)	1135 (23.4)	0.35
Cardiac care	57 (1.1)	39 (0.8)	0.066
Neurology	104 (2.1)	88 (1.8)	0.248
Plastic surgery	97 (1.9)	56 (1.1)	0.001
Orthopedic	20 (0.004)	21 (0.4)	0.876

### Pharmaceutical unit reduction

A total number of 13,821 medications were used in the pre-intervention period. After guideline implementation, this rate decreased to 6539. The reduction in requests (50.76%) was statistically significant (*P* < 0.001). In line with this, the total number of administered albumin and IV pantoprazole decreased significantly after the intervention. In contrast, the number of administered IVIG as well as its direct cost was significantly higher in the post-intervention compared to pre-intervention phase (Table 2).

**Table 2:** Comparison of monthly consumption of discussed medications and their relative monthly costs between pre-intervention and post-intervention groups

Variable	Medication	Pre-intervention group (*n* = 4946)	Post-intervention group (*n* = 4895)	Portion of reduction	*p*
Number of medications used	Albumin	5636	2771	50.83%	< 0.001
	Pantoprazole	7623	3027	60.29%	< 0.001
	IVIG	562	741	- 31.85%^a^	< 0.001
Cost (USD) [mean ± SD or median (IQR)]	Albumin	245,885 ± 18,616	108,689 ± 10,429	55.8%	< 0.0001
	Pantoprazole	62,399 (7769)	10,036 (1759)	83.92%	0.004
	IVIG	69,234 ± 17,182	97,565 ± 19,164	−40.9%^a^	0.022

^a^Negative value means that IVIG cost and number of use increased in the post-intervention phase compared to the pre-intervention period

### Pharmaceutical cost reduction

Compared to the pre-intervention period, the direct costs of albumin and IV pantoprazole significantly reduced by 55.8% and 83.92%, respectively in the post-intervention period. In contrast, the mean ± SD of direct cost of IVIG per month in the post-intervention phase ($97,565 ± 19,164) was significantly higher (*P* = 0.022) than the pre-intervention period ($69,234 ± 17,182) (Table 2). The mean ± SD of total net direct cost of all studied medications per month decreased from $139,102 ± 91,342 in the pre-intervention period to $61,382 ± 49,514 in the post-intervention period. In other words, the monthly direct cost of these three medications reduced by more than half (55.88% equal to $77,720) after guideline implementation, which was statistically significant (*P* < 0.0001). The annual net cost-saving for albumin, IV pantoprazole, and IVIG was estimated to be approximately $932,640.

### Alternative medication costs

In order to assess the changes in cost associated with switching from the studied medications to other agents after guideline implementation, the direct costs of Amino Acid 5% and 10% (as alternatives to albumin in case of parenteral nutrition), oral pantoprazole, oral omeprazole and intravenous ranitidine (as alternatives for IV pantoprazole in case of stress-related mucosal damage prophylaxis) and parenteral methylprednisolone sodium succinate, methylprednisolone acetate, dexamethasone, betamethasone, and rituximab (as alternatives to IVIG in case of autoimmune diseases) were compared between pre- and post- intervention periods.

The mean ± SE monthly direct cost of Amino Acid 5 and 10% was comparable between the pre- and post- intervention periods ($9331 ± 5252 versus $9730 ± 5994; *P* = 0.962). Similarly, the median total direct cost per month for oral pantoprazole, oral omeprazole, and intravenous ranitidine did not differ significantly (*P* = 0.827) between the pre-intervention ($3782) and post-intervention ($4314) phases. In contrast, the median direct cost per month for parenteral corticosteroids and rituximab in the post-intervention phase ($924.92 and $18,280.26, respectively) was significantly higher (*P* < 0.001 for both medication classes) than the pre-intervention phase ($797.47 and $10,189.86, respectively).

### Causes of medication request disapproval

Table 3 lists the causes of disapproved requests for albumin and IV pantoprazole in the post-intervention period. Management of edema in patients with serum albumin level above 2 g/dL (24.58%) was the most common inappropriate use of albumin in the post-intervention period, followed by administration as a component of parenteral nutrition (19.87%). Regarding IV pantoprazole, the three most frequent causes of disapproval were the ability to tolerate oral medications (53.02%), presence of only one minor risk factor for prophylaxis of stress-related mucosal damage (24.78%), and acute pancreatitis treatment (6.25%). IVIG requests were not approved by the pharmaceutical care unit in only five cases, including non-refractory sepsis (*n* = 2), Devic’s disease (*n* = 2), and idiopathic thrombocytopenic purpura in a patient with platelet count above 30,000/mm^3^ without active bleeding or planning for an emergent procedure (*n* = 1).

**Table 3:** Causes of albumin and pantoprazole disapproval within the intervention period

Albumin		Pantoprazole	
Cause of disapproval	Number (%)	Cause of disapproval	Number (%)
Management of edema in patients with serum albumin level above 2 g/dL	73 (24.58)	Ability to tolerate an oral PPI	246 (53.02)
A component of parenteral nutrition	59 (19.87)	Presence of only one minor risk factor for stress-related mucosal damage prophylaxis	115 (24.78)
Monotherapy of edema without a loop diuretic	36 (12.12)	Treatment of acute pancreatitis	29 (6.25)
Therapeutic paracentesis with ascitic fluid removal less than 5 L	21 (7.07)	Treatment of acute cholangitis or cholecystitis	21 (4.53)
Monotherapy of hepatorenal syndrome without a vasoactive agent	17 (5.72)	Gastric and/or pancreas cancer	17 (3.66)
Prolonged prevention (more than 2 weeks) of cerebral vasospasm in patients with subarachnoid hemorrhage	11 (3.7)	Lower GI obstruction or bleeding	15 (3.23)
Non-ARDS condition	3 (1.01)	Non-refractory (mild) dyspepsia	9 (1.94)
Others (no specific explanation)	42 (14.14)	Others (no specific explanation)	12 (2.59)

*ARDS* Acute respiratory distress syndrome, *PPI* Proton pump inhibitor, *GI* Gastrointestinal

### Clinical outcomes

The median IQR for LOS, as well as all-cause in-hospital mortality rate, was significantly higher in the post-intervention phase than the pre-intervention phase (*P* < 0.001 and *P* = 0.043, respectively). For each medication separately, the mortality rate of patients who were prescribed pantoprazole IV was significantly higher (*P* = 0.033) in the pre-intervention (9.5%) than the post-intervention phase (9.2%) (Table 4).

**Table 4:** Comparison of clinical outcomes between pre- and post-intervention groups

Clinical outcomes	Pre-intervention group (*n* = 4946)	Post-intervention group (*n* = 4895)	*p*
LOS, in days, (Median, IQR)	6 (9)	7 (11)	< 0.001
Hospital discharge, *n* (%)	4209 (85.1)	4093 (83.6)	0.043
All-cause in-hospital mortality, *n* (%)			
Albumin	250 (5.1)	316 (6.5)	0.994
Pantoprazole	470 (9.5)	448 (9.2)	0.033
IVIG	17 (0.3)	38 (0.8)	0.761
Total	737 (14.9)	802 (16.4)	0.043

*LOS* Length of stay, *IQR* Interquartile range

The results of univariate and multivariate logistic regression analyses regarding mortality rate in all patients (sum of pre- and post-intervention phases) are demonstrated in Table 5. According to the univariate analysis, type of ward (*P* < 0.001), age (*P* < 0.001), and intervention (*P* = 0.043) were significantly associated with mortality. Similarly, these variables remained statistically significant in the multivariate logistic regression model.

**Table 5:** Univariate and multivariate logistic regression models of the possible association between mortality (as dependent variable) and demographic as well as clinical features (as independent variables) of all patients in pre- and post-intervention groups

Variable	Mortality (*n* = 1539)	Discharged (*n* = 8302)	Univariate analysis		Multivariate analysis	
			OR (95% CI)	*p*	OR (95% CI)	*p*
Age (years)						
Mean ± SD	50.04 ± 26.66	43.32 ± 25.99	1.010 (1.008–1.012)	< 0.001	1.008 (1.006–1.010)	< 0.001
Sex (%)						
Male	909 (59.06)	4943 (59.54)	1.020 (0.913–1.139)	0.727	**–**	**–**
Female	630 (40.94)	3359 (40.46)				
Type of ward (%)						
Internal^a^	552 (35.87)	1343 (16.18)	0.873 (0.856–0.890)	< 0.001	0.880 (0.864–0.898)	< 0.001
Surgical^b^	160 (10.39)	1480 (17.83)				
Critical care^c^	346 (22.48)	1424 (17.15)				
Emergency	209 (13.58)	2106 (25.37)				
Pediatrics	272 (17.67)	1949 (23.48)				
Admission diagnosis (%)						
Gastrointestinal diseases	437 (28.39)	2775 (33.43)	0.999 (0.967–1.01)	0.298	**–**	**–**
Hematologic-oncologic diseases	255 (16.57)	1134 (13.66)				
Infectious diseases	241 (15.66)	905 (10.9)				
Cardiovascular diseases	164 (10.66)	806 (9.71)				
Neurologic diseases	133 (8.64)	868 (10.46)				
Kidney and urinary tract diseases	123 (7.99)	709 (8.54)				
Lung diseases	85 (5.52)	376 (4.53)				
Metabolic diseases	27 (1.75)	152 (1.83)				
Traumatic diseases	21 (1.36)	136 (1.64)				
Autoimmune disease	14 (0.91)	151 (1.82)				
Others^d^	39 (2.53)	290 (3.49)				
Intervention phase (%)						
Pre	737 (47.89)	4209 (50.69)	1.119 (1.004–1.248)	0.043	1.128 (1.009–1.262)	0.034
Post	802 (52.11)	4093 (49.3)				

^a^Including general, nephrology, gastrointestinal, cardiac, and neurology wards

^b^Including general surgery, cardiac surgery, neurosurgery, urology, plastic surgery, and orthopedic wards

^c^Includingintensive care and cardiac care units

^d^Including rheumatologic diseases, skin/soft tissue diseases, electrolyte disorders, immune deficiency, and diseases of prematurity

*OR* Odds ratio, *CI* Confidence interval

## Discussion

### Pharmaceutical expense reduction

The intervention by the pharmaceutical care unit via implementing clinical guidelines in a referral hospital in Southwest of Iran significantly decreased the direct cost of albumin and IV pantoprazole, but not IVIG. Although evidence-based medicine supports clinical effectiveness and theoretical as well as pharmacological benefits of albumin, IV pantoprazole, and IVIG in certain conditions, they can be overused or their usage pattern may be inappropriate.

In the past three decades, clinical studies from different countries have indicated that at least 50% to more than 90% of albumin prescriptions are inappropriate [10–13]. Overuse of albumin can be challenging for healthcare systems due to its high cost, limited availability, and potential risk of pathogen transmission [10]. Regarding the costs, a report by the Iranian Food and Drug Organization of Health Ministry indicated that 472,089 vials of albumin 20% have been used within the first 9 months of 2008, which amounts to $21,600,000 (13). By implementing clinical guidelines in our center, the number of administered vials of albumin and its direct cost significantly reduced by 50.83% and 55.8%, respectively. In line with these data, use of albumin guidelines in a surgical intensive care unit (ICU) of a tertiary teaching hospital in the Unites States resulted in the significant reduction of albumin use (54%) and substantial cost-saving (56%) [14].

IV pantoprazole overuse, besides its high cost, can be associated with life-threatening side effects (e.g., *Clostridium difficile* diarrhea) and drug interactions (e.g., clopidogrel) [15]. Batuwitage et al. reported that proton pump inhibitor (PPI) use was inappropriate in 54% of its recipients in general medical wards of the UK [16]. Although the rate of inappropriate use of IV pantoprazole was unknown in the pre-intervention period in our study, clinical guideline implementation was associated with a significant reduction in the number of administrations by 60.29% and direct cost by 83.92%.

Reduction in PPI use through implementation of appropriate guidelines has been also reported by other researchers. For example, Van Vliet et al. in the Netherlands demonstrated that guideline implementation for PPI prescription was associated with significantly fewer patients starting on PPIs during their hospitalization in two pulmonary medicine wards, compared to the control group (13% versus 21%) [17]. A recent study on implementation of pharmaceutical practice guidelines for three costly medications at a tertiary hospital in Iran resulted in a significant reduction in prescriptions of albumin (36%) and IV pantoprazole (40%) [18].

The comparable direct cost of possible alternative medications for albumin (Amino Acid 5% and 10%) and IV pantoprazole (oral omeprazole, oral pantoprazole, and IV ranitidine) between the pre-intervention and post-intervention phases demonstrated that our intervention and switch in use from the studied medications to the alternatives did not result in an increase in the costs. However, in the post-intervention period, the monthly direct costs of parenteral corticosteroids and rituximab, as IVIG alternatives, were significantly higher than the pre-intervention phase. This may be mostly related to the ability of pharmacies to provide these drugs, and subsequently, the increase in their demand during the post-intervention phase. In this regard, higher consumption and direct cost of IVIG in the post-intervention period is another reason to confirm that switch in use from IVIG to its alternatives is not the cause of increase in their cost within the post-intervention phase.

Despite its high cost, global limited sources, several potential adverse effects (e.g., acute kidney injury and hypersensitivity reactions), and documented cases of hepatitis C transmission [19], the list of possible indications and amount of consumed IVIG have grown rapidly worldwide [20]. The annual global demand for IVIG has shown an increase from 7.4 to 55.0 metric tons from 1984 to 2004 [20]. To the best of our knowledge, there is no official national report or published literature on the consumption rate or cost of IVIG in Iran. Our current intervention failed to result in a significant reduction in the administration rate or direct cost of IVIG. This may be due to two major reasons:

First, completing the indication forms by physicians cannot be the sole effective approach for improving the pattern of IVIG use. More than 50 known off-label indications, patients’ critical clinical conditions, pharmacists’ insufficient knowledge regarding patients’ conditions, and their reliance on diagnosis notes in the patients’ medical charts may explain this result. In this regard, Frayha et al. demonstrated that concurrent action plans, including guideline dissemination to all healthcare teams along with indication form use, were associated with the improved utilization pattern, as well as a 14% decrease ($41,000) in the expenditure of IVIG [21]. In keeping with these data, use of IVIG utilization management tools, including distribution of IVIG guidelines among specialists, development of preprinted IVIG order forms, and IVIG dose adjustments based on trough IgG levels for physicians resulted in a total cost saving of $3,038,056 in 2 years in four Canadian Atlantic Provinces [22].

Second, there was a relative shortage of IVIG formulation in the pre-intervention phase, compared to the post-intervention period. In addition, financial resources for providing IVIG were higher and more available during the post-intervention phase in our center due to the start of the Health Revolution Program since May 2013 in Iran. This issue may have resulted in the increased demand for IVIG in the later phase of the study.

### Clinical outcomes

Although our pharmacist-based intervention significantly decreased the total direct cost of the studied medications, it was associated with an elevated all-cause in-hospital mortality rate (14.9% versus 16.4% in the pre- versus post-intervention periods), which was statistically significant. This remained statistically significant even after adjusting for confounding factors, including clinical and demographic characteristics of the population. Besides our intervention, advanced age and internal ward admission were also significantly associated with all-cause in-hospital mortality.

The sub-group analysis revealed that the in-hospital mortality rates were higher in patients receiving albumin and IVIG during the post-intervention phase in comparison to the pre-intervention phase (1.4% and 0.5%, respectively). However, these differences were not statistically significant; also, these differences had no clinical relevance. In line with our data, a two-year evidence-based sequential multifaceted intervention was done on the use of albumin in eight ICUs in the USA. The intervention was associated with the estimated total cost saving of $2.5 million without any significant difference in ICU and in-hospital mortality rates between the baseline and post-intervention [23].

The case appears to be somewhat different for IV pantoprazole, compared to albumin and IVIG in our cohort. The sub-analysis implicated that mortality rate was slightly higher (0.3%) in the pre-intervention period, compared to the post-intervention period. Although this rate was statistically significant, it was unlikely to be therapeutically important. Since stress-related mucosal damage prophylaxis is one of the major indications of IV pantoprazole, it seems crucial to determine the incidence of upper GI bleeding episodes in both pre- and post-intervention periods as a more direct and relevant clinical outcome index. However, extracting these data from the medical records of patients or the Hospital Information System in our center was not feasible during this study.

At least two studies by Van Vliet et al. [17] and Mahmoudi et al. [18] demonstrated that guideline implementation for PPI prescription did not increase the risk of upper GI disorders and GI bleeding, respectively. Finally, a systematic review of 20 randomized clinical trials in adult ICU patients (*n* = 1971) regarding stress ulcer prophylaxis showed no significant difference between stress ulcer prophylaxis and placebo (no prophylaxis) in terms of mortality and GI bleeding [24].

The average LOS in hospital, as another studied clinical outcome in our investigation, was one day longer in the post-intervention period in comparison with the pre-intervention phase. Although the difference was statistically significant, this should not be essentially interpreted as the direct effect of our intervention on prolonging LOS in hospital. In this regard, Freitas et al. examined variables related to high LOS outliers in nearly nine million inpatient episodes in the Portuguese National Health System. They demonstrated that different variables, such as teaching hospitals (versus non-teaching hospitals), increased age, emergent surgeries (versus planned or elective surgeries), and number of comorbidities, were significantly associated with increased LOS [25].

Data regarding the number of comorbidities and type of surgery were not available in our population to evaluate their possible confounding effects on LOS. Furthermore, patients in the pre- and post-intervention periods were not matched in terms of age and hospital wards. In contrast to our findings, the mean LOS was comparable before and after guideline implementation for three costly medications (albumin, IV pantoprazole, and enoxaparin) in a teaching hospital in Iran [18]. Differences in the complexity of both hospitals and patients, as major determinants of LOS, can partially explain LOS disparities in our study and the study by Mahmoudi and colleagues.

### Inappropriate use

The two most common inappropriate uses of albumin in the post-intervention period were management of edema in patients without severe hypoalbuminemia (24.58%) and a component of parenteral nutrition (19.87%). Similarly, Jahangard-Rafsanjani et al. reported that 46.6% of albumin administrations in a healthcare setting in Tehran were for nutritional support [13]. Tanzi et al. in a study on 1672 patients from 53 different healthcare facilities in the USA showed that all 142 indications of albumin for individuals with serum albumin levels below 2 g/dL were inappropriate [12]. It has been demonstrated that enteral and/or parenteral nutrition with amino acids, along with adequate calorie intake rather than albumin, can improve serum albumin level in malnourished patients [10].

Regarding IV pantoprazole, oral tolerance in the absence of enteral tube and lack of indications for prophylaxis of stress-related mucosal damage are the two leading causes of disapproving the medication requests in the intervention phase of our study. Accordingly, there is a misconception by a number of physicians that parenteral PPIs may be more efficacious than their oral formulations. However, no head-to-head or comparative clinical studies have confirmed this idea. Nevertheless, since PPIs degrade in acidic environments and are formulated in a delayed-release formulation, they should not be crushed for administration through the enteral feeding tube [26]. In the Netherlands, preventing medication-associated complications in two pulmonary medicine wards, including NSAIDs, corticosteroids, and antibiotics, was the most common reason for PPI use [27].

In contrast to albumin and IV pantoprazole, inappropriate use of IVIG in our cohort in the post-intervention period was only limited to five cases. Similarly, Constantine et al. demonstrated that after implementing IVIG guidelines and feedback reports in the Atlantic Canada, IVIG utilization for labeled indications remained unchanged (37.1% and 36.1% in the baseline and post-intervention, respectively). The rate of unlabeled but potentially indicated use of IVIG increased from 52.4% at baseline to 58.1% in the implementation phase [22].

 A four-year experience in an academic center in Italy implicated that the majority of IVIG uses in neurological and neuromuscular disorders were identified to be either recommended (60.4%) or reasonable (25.6%) [28]. Therefore, according to the findings of our cohort and other relevant studies, as well as suggestions by Pendergrast et al., guideline implementation seems unlikely to have significant decreasing effects on the total amount of IVIG consumption in academic and teaching environments [20].

Conducting clinical trials regarding IVIG indications, which have been only proposed in case reports and uncontrolled case series, using effective and cheaper treatments rather than IVIG, and developing a multispecialty team along with multiple-level surveillance for evaluating and approving IVIG indications can be taken into account as effective approaches to improve the usage pattern of this highly popular and commonly prescribed, but limited-source and costly medication.

## Conclusion

The current study demonstrated that a pharmacist-based guideline implementation during a six-month period on 4896 patients significantly reduced the monthly direct cost of albumin, IV pantoprazole, and IVIG by $77,720 in a referral clinical setting in Iran. However, this reduction was along with a minor and clinically negligible, but statistically significant increase in all-cause in-hospital mortality and hospital LOS. Furthermore, our intervention including switching the studied medications to their alternatives did not result in an increase in pharmaceutical costs. In contrast to albumin and IV pantoprazole, our intervention failed to significantly reduce the administration rate or direct cost of IVIG. Multidisciplinary strategies, such as guideline dissemination, educating physicians regarding proper indications of drugs, and clinical guideline implementation supervised by pharmaceutical care units appear to be more effective in improving the usage pattern and reducing pharmaceutical costs for medications with several labeled and off-label uses, such as IVIG.

## Additional file

Additional file 1: Indication checklists for albumin, IVIG, and iv pantoprazole. (ZIP 65 kb)

## Abbreviations

ARDS: Acute Respiratory Distress Syndrome
GI: Gastrointestinal
ICU: Intensive Care Units
IQR: Interquartile Range
IV: Intravenous
IVIG: Intravenous Immune Globulin
LOS: Length of Stay
PPI: Proton Pump Inhibitors
