# Supplementary material for: Clinical and economical impacts of guideline implementation by the pharmaceutical care unit for high cost medications in a referral teaching hospital
Source: BMC Health Serv Res. 2018 Oct 24;18:815. doi: 10.1186/s12913-018-3627-3 (PMC6201544; doi:10.1186/s12913-018-3627-3)
Supplement: Supplementary file 1 — Indication checklists for albumin, IVIG, and iv pantoprazole. (ZIP 65 kb) [file 12913_2018_3627_MOESM1_ESM.zip › Albumin indication checklistR5.docx]

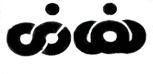


| **Patient name:** | **U.No:** | | **Ward:** | |  |  |  |
| --- | --- | --- | --- | --- | --- | --- | --- |
| **Time & Date of filling out the form:** | | **Serum Alb:** | | |  |  |  |
| **Indication** | **Note** | | | **√** |  |  |  |
| **Paracentesis** | **5 g of albumin/L ascitic fluid removed, after paracentesis of volumes > 5 L** | | |  |  | |  |
| **Therapeutic plasmapheresis** | **For exchanges of > 20 mL/kg in one session or > 20 mL/kg/week in more than one session alone or in combination with crystalloid fluids** | | |  |  | |  |
| **Spontaneous bacterial peritonitis** | **In combination with antibiotics if the creatinine is >1 mg/dL, the blood urea nitrogen is >30 mg/dL , or the total bilirubin is >4 mg/dL** | | |  |  | |  |
| **Major surgery** | **Serum albumin <3 g/dL before or early after surgery** | | |  |  | |  |
| **Cirrhosis of the liver with refractory ascites** | **Serum albumin <3 g/dL** | | |  |  | | |
| **Acute lung injury (ALI )or acute respiratory distress syndrome (ARDS)** | **Alone or in combination with furosemide** | | |  |  | |  |
| **Aneurysmal subarachnoid hemorrhage (SAH)** | **1.25 mg/kg/day for up to 2 weeks** | | |  |  | |  |
| **Hemorrhagic shock** | **Only in the case of lack of response to crystalloids** | | |  |  |  |  |
| **Hepatorenal syndrome** | **In association with vasoactive agents** **(e.g.,** **Norepinephrine, Midodrine)** | | |  |  |  |  |
| **Nephrotic syndrome** | **In combination with a loop diuretic if serum albumin <3 g/dL or alone if serum albumin <2 g/dL** | | |  |  |  |  |
| **Burns** | **In the case of burns of > 30% body surface area, after the first 24 hours** | | |  |  |  |  |
| **Albumin Order:**  **Dose:**  **Interval:**  **Start date:**  **Discontinuation date:** | | | | |  |  |  |
| **Physician comments:**  **Physician date & signature:** | | | | |  |  |  |
| **Pharmacist comments:**  **Pharmacist date & signature:** | | | | |  |  |  |
| **Indication approved □ Indication not approved□** | | | | |  |  |  |

**Albumin indication checklist**
