# Supplementary material for: Clinical and economical impacts of guideline implementation by the pharmaceutical care unit for high cost medications in a referral teaching hospital
Source: BMC Health Serv Res. 2018 Oct 24;18:815. doi: 10.1186/s12913-018-3627-3 (PMC6201544; doi:10.1186/s12913-018-3627-3)
Supplement: Supplementary file 1 — Indication checklists for albumin, IVIG, and iv pantoprazole. (ZIP 65 kb) [file 12913_2018_3627_MOESM1_ESM.zip › Intravenous Pantoprazole indication checklistR5.docx]

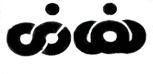


| **Patient name:** | **U.No:** | | **Ward:** | | | |  |  |
| --- | --- | --- | --- | --- | --- | --- | --- | --- |
| **Time & Date of filling out the form:** | | **Platelet count: PTT: INR:** | | | | |  |  |
| **◼ Stress ulcer prophylaxis** | | | | |  | |  |  |
| **- Major risk factors** | | | | | | |  |  |
| - **Coagulopathy (Plt<50000 or INR>1.5 or aPTT>2 times the control value)** | | | |  | | |  |  |
| - **Hx of GI ulceration or bleeding within 1year of admission** | | | |  | | |  |  |
| - **Glasgow coma scale of <8** | | | |  | | |  |  |
| - **Thermal injury to > 35% of body surface area** | | | |  | | |  |  |
| - **Partial hepatectomy** | | | |  | | |  |  |
| - **Multiple trauma** | | | |  | |  |  | |
| - **Hepatic or renal transplantation** | | | |  | | |  |  |
| - **Spinal cord injury** | | | |  | | |  |  |
| - **Hypoperfusion (sepsis, shock, multi organ failure or vasoactive therapy)** | | | |  | | |  |  |
| - **Mechanical ventilation for >48 hours** | | | |  | | |  |  |
| **- Minor risk factors** | | | | | | |  |  |
| - **Mild to moderate brain injury (GCS > 8)** | | | |  | | |  |  |
| - **Occult bleeding lasting > 6days** | | | |  | | |  |  |
| - **NSAID use** | | | |  | | |  |  |
| - **Clopidogrel use** | | | |  | | |  |  |
| - **High dose corticosteroid (>250 mg of hydrocortisone or equivalent daily)** | | | |  | | |  |  |
| **◼ Acute upper gastrointestinal bleeding** | | | |  | | |  |  |
| **◼ Refractory peptic ulcer disease** | | | |  | | |  |  |
| **◼ Refractory gastroesophageal reflux disease** | | | |  | | |  |  |
| **Pantoprazole Order:**   - **Dose:** - **Interval:** - **Start date:** - **Discontinuation date:** | | | | | | |  |  |
| **Physician comments:**  **Physician date & signature** | | | | | | |  |  |
| **Pharmacist comments:**  **Pharmacist date & signature** | | | | | | |  |  |
| **Indication approved □ Indication not approved □** | | | | | | |  |  |

**Intravenous Pantoprazole Order Form**
