# Supplementary material for: Clinical and economical impacts of guideline implementation by the pharmaceutical care unit for high cost medications in a referral teaching hospital
Source: BMC Health Serv Res. 2018 Oct 24;18:815. doi: 10.1186/s12913-018-3627-3 (PMC6201544; doi:10.1186/s12913-018-3627-3)
Supplement: Supplementary file 1 — Indication checklists for albumin, IVIG, and iv pantoprazole. (ZIP 65 kb) [file 12913_2018_3627_MOESM1_ESM.zip › IVIG indication checklistR5.docx]

**
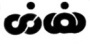
**

**Immune globulin intravenous (IVIG) indication checklist**

| **Patient name:** | **U.No:** | **Ward:** | |
| --- | --- | --- | --- |
| **Time & Date of filling out the form:** | | | |
| **Indication** | | | √ |
| Prevention of bacterial infections in patients with hypogammaglobulinemia and/or recurrent bacterial infections associated with B-cell **chronic lymphocytic leukemia (CLL) or multiple myeloma** | | |  |
| Prevention of bacterial infections in **allogeneic recipients** with hypogammaglobulinemia (IgG<400 mg/dL) at <100 days post transplant | | |  |
| Treatment of **chronic inflammatory demyelinating polyneuropathy (CIDP)** | | |  |
| Treatment of **hyperhemolytic crisis** resistant to other agents (e.g., corticosteroids) | | |  |
| Treatment of **neonatal jaundice** secondary to Rh or ABO incompatibility-associated hemolytic disease | | |  |
| Treatment of **multifocal motor neuropathy (MMN)** | | |  |
| Pre- and post-exposure prophylaxis against **hepatitis A** | | |  |
| Post-exposure prophylaxis against **measles** in infants younger than 12 months, in pregnant women in whom there is no evidence of measles immunity and in severely immunocompromised persons | | |  |
| Post-exposure prophylaxis against **rubella** in pregnant mothers who do not choose a therapeutic abortion | | |  |
| Post-exposure prophylaxis against **varicella** exposure if Varicella-Zoster immune globulin is not available | | |  |
| Treatment of **primary humoral immunodeficiency syndromes** (congenital agammaglobulinemia, severe combined immunodeficiency syndromes, common variable immunodeficiency, X-linked immunodeficiency, Wiskott-Aldrich syndrome) | | |  |
| Treatment of **platelet alloimmunization** or **post transfusion purpura** | | |  |
| Treatment of resistant acquired **pure red cell aplasia** | | |  |
| Treatment of **Lambert Eaton syndrome** | | |  |
| Treatment of **Intractable childhood epilepsy** | | |  |
| Treatment of **Orseclum myoclonus syndrome** | | |  |
| Treatment of severe **acute disseminated encephalomyelitis (ADEM)** not responsive to glucocorticoids | | |  |
| Treatment of auto-immune **anti-NMDA receptor encephalitis** | | |  |
| Treatment of **Stiff-person syndrome** not responsive to or unable to tolerate glucocorticoids | | |  |
| Treatment of acute and chronic **idiopathic thrombocytopenic purpura (ITP)** | | |  |
| Treatment of refractory or relapsing **thrombotic thrombocytopenic purpura-hemolytic uremic syndromes (TTP-HUS)** | | |  |
| Prevention of coronary artery aneurysms associated with **Kawasaki syndrome** in combination with aspirin | | |  |
| Treatment of **acute humoral rejection** in kidney transplant patients in combination with plasmapheresis | | |  |
| Desensitization of **highly-sensitized patients** (PRA > 20%) awaiting renal transplantation | | |  |
| Treatment of **BK nephropathy** in solid organ transplant patients | | |  |
| Treatment of **severe IgA nephropathy** | | |  |
| Treatment of **Guillain-Barre syndrome** | | |  |
| Treatment of **Myasthenia gravis** | | |  |
| Treatment of **HIV-associated idiopathic thrombocytopenia purpura**, serious infections, or anemia due to parvovirus B19 infection | | |  |
| Treatment of **cytomegalovirus (CMV) diseases (e.g., pneumonia, retinitis)** in combination with ganciclovir in HIV or transplant patients | | |  |
| Treatment of biopsy-confirmed or highly suspected **acute myocarditis** in children | | |  |
| Treatment of **relapsing/remitting multiple sclerosis (MS)** when other therapies cannot be used | | |  |
| Treatment of **systemic dermatomyositis**, or **polymyositis** refractory to glucocorticoids | | |  |
| Treatment of refractory **systemic vasculitis** | | |  |
| Treatment of refractory **systemic lupus erythematousus (SLE)** | | |  |
| Treatment of adult-onset **still disease** | | |  |
| Treatment of refractory **catastrophic antiphospholipid syndrome** | | |  |
| Treatment of early phases of severe **Stevens-Johnson syndrome** or **Toxic epidermal necrolysis** | | |  |
| Treatment of **severe sepsis** in critically ill patients in combination with broad spectrum antibiotics | | |  |
| Treatment of **post-transplantation lymphoproliferative disorder (PTLD)** in combination with chemotherapeutic agents | | |  |
| Prevention of **recurrent spontaneous abortion** | | |  |
| Treatment of **birdshot retinochoroidopathy** (bilateral autoimmune posterior uveitis) | | |  |
| **IVIG Order: -**  **Dose & Interval & Route of administration:**  **Start & Discontinuation date:** | | |  |
| **Physician comments:**  **Physician date & signature:** | | |  |
| **Pharmacist comments:**  **Pharmacist date & signature:** | | |  |
| **Indication approved □ Indication not approved □** | | |  |
